# Type II Toxin-Antitoxin Systems and Persister Cells

**DOI:** 10.1128/mBio.01574-18

**Published:** 2018-09-25

**Authors:** David W. Holden, Jeff Errington

**Affiliations:** a MRC Centre for Molecular Bacteriology and Infection, Imperial College London, London, United Kingdom; b Centre for Bacterial Cell Biology, Institute for Cell and Molecular Biosciences, Newcastle University, Newcastle upon Tyne, United Kingdom; National Institute of Child Health and Human Development (NICHD)

**Keywords:** *E. coli*, bacteriophage, persisters, toxin-antitoxin

## LETTER

Last year, Gerdes and colleagues published a paper (1) describing experiments that failed to support earlier work from their group (2, 3) which had implicated 10 type II toxin-antitoxin (TA) systems in the formation of antibiotic-tolerant Escherichia coli K-12 persister cells. The problem apparently arose as a result of contamination by and activation of the cryptic bacteriophage Φ80 in mutant strains lacking TA genes. A more recent paper by Goormaghtigh et al. (4) confirms and extends this reappraisal by providing evidence that an independently constructed E. coli K-12 mutant strain lacking the 10 type II TAs and free of phage contamination produced levels of persisters similar to those of wild-type bacteria after exposure to antibiotics (4). In addition, this work questions the validity of TA::green fluorescent protein (GFP) transcriptional reporter fusions (3). Since the possible link between TA systems and the persister phenotype is being studied in many laboratories, these corrections are both important and salutary.

However, we highlight what seem to us to be some overstatements and factual inaccuracies in the highly critical paper of Goormaghtigh et al. (4).

First, the authors state that “results obtained with an independently constructed *Δ10TA* strain do not support a role for TA systems in persistence….” However, their polymutant strain was analyzed only at mid-exponential growth phase in “optimally balanced” medium. It is not clear whether the relevant TA systems are physiologically active in these conditions, and the mutant needs to be subjected to further phenotypic analysis (e.g., following physiological stress) before general conclusions can be drawn about the involvement of TA systems in E. coli K-12 persister formation. It is noteworthy that a study from another group showed that a strain lacking one of the type II toxin genes mutated in the *Δ10TA* strain (*yafQ*) had a very strong defect in antibiotic tolerance when grown as a biofilm (5).

Second, the authors state that “The model linking TA systems and persistence to antibiotics had a major impact in the microbiology community as a whole. Recently, this model was invalidated….” The purported invalidation relates only to nonstressed E. coli K-12. Evidence for the involvement of TA systems in persister formation has been obtained for several other bacteria, including uropathogenic E. coli (6), *Burkholderia* (7), and *Salmonella* (8–10).

Third, the authors state that “The model linking TA modules and persistence initially stemmed from observations made by the K. Gerdes lab that successive deletions of 10 type II TA systems… progressively decreased the level of persistence to antibiotics.” In fact, this model goes back over 30 years to a phenotypic analysis of the *hipA7* mutant that displays enhanced levels of persister formation (11). Furthermore, forced overexpression of the toxin RelE (12) or MazF (13, 14) in E. coli led to significant increases in persister cells. These papers therefore provide additional evidence linking TA systems to persisters.

Scientific research is inherently error-prone: in experimental design, execution, and interpretation. What matters is not error *per se* but recognition of it. We commend Kenn Gerdes and his group for their scientific probity in setting the record straight (1, 15). Clearly, further work is needed to establish the relative contributions of TA systems to persister formation in E. coli K-12 strains and other bacteria.
